# Functional analysis of liverworts in dual symbiosis with Glomeromycota and Mucoromycotina fungi under a simulated Palaeozoic CO_2_ decline

**DOI:** 10.1038/ismej.2015.204

**Published:** 2015-11-27

**Authors:** Katie J Field, William R Rimington, Martin I Bidartondo, Kate E Allinson, David J Beerling, Duncan D Cameron, Jeffrey G Duckett, Jonathan R Leake, Silvia Pressel

**Affiliations:** 1 School of Biology, Faculty of Biological Sciences, University of Leeds, Leeds, UK; 2 Department of Life Sciences, Imperial College London, London, UK; 3 Jodrell Laboratory, Royal Botanic Gardens, Kew, UK; 4 Department of Life Sciences, Natural History Museum, London, UK; 5 Department of Animal and Plant Sciences, Western Bank, University of Sheffield, Sheffield, UK

## Abstract

Most land plants form mutualistic associations with arbuscular mycorrhizal fungi of the Glomeromycota, but recent studies have found that ancient plant lineages form mutualisms with Mucoromycotina fungi. Simultaneous associations with both fungal lineages have now been found in some plants, necessitating studies to understand the functional and evolutionary significance of these tripartite associations for the first time. We investigate the physiology and cytology of dual fungal symbioses in the early-diverging liverworts *Allisonia* and *Neohodgsonia* at modern and Palaeozoic-like elevated atmospheric CO_2_ concentrations under which they are thought to have evolved. We found enhanced carbon cost to liverworts with simultaneous Mucoromycotina and Glomeromycota associations, greater nutrient gain compared with those symbiotic with only one fungal group in previous experiments and contrasting responses to atmospheric CO_2_ among liverwort–fungal symbioses. In liverwort–Mucoromycotina symbioses, there is increased P-for-C and N-for-C exchange efficiency at 440 p.p.m. compared with 1500 p.p.m. CO_2._ In liverwort–Glomeromycota symbioses, P-for-C exchange is lower at ambient CO_2_ compared with elevated CO_2_. No characteristic cytologies of dual symbiosis were identified. We provide evidence of a distinct physiological niche for plant symbioses with Mucoromycotina fungi, giving novel insight into why dual symbioses with Mucoromycotina and Glomeromycota fungi persist to the present day.

## Introduction

Symbioses with soil fungi have existed since plants first began to colonize the Earth's land masses (Redecker *et al.*, 2000; Redecker and Raab, 2006; Smith and Read, 2008) and are thought to have played a key role in establishing terrestrial ecosystems (Pirozynski and Malloch, 1975; Malloch *et al.*, 1980). There are numerous lines of supporting evidence for this view, including plant and fungal fossils (Stubblefield *et al.*, 1987; Remy *et al.*, 1994; Taylor *et al.*, 1995) and molecular data (Simon *et al.*, 1993; Redecker *et al.*, 2000; Redecker and Raab, 2006). Recent studies of ultrastructure (Pressel *et al.*, 2010) and plant–fungal physiology of early-diverging extant land plant lineages (Field *et al.*, 2012, 2015a) provided new insights into the structure–function relationships of non-vascular plants and their symbiotic fungi. Until recently, the fungal associates of the earliest branching plant lineages have been assumed to be members of the arbuscular mycorrhiza-forming clade of fungi, the obligately biotrophic Glomeromycota that lack saprotrophic capabilities.

**Figure 1:**
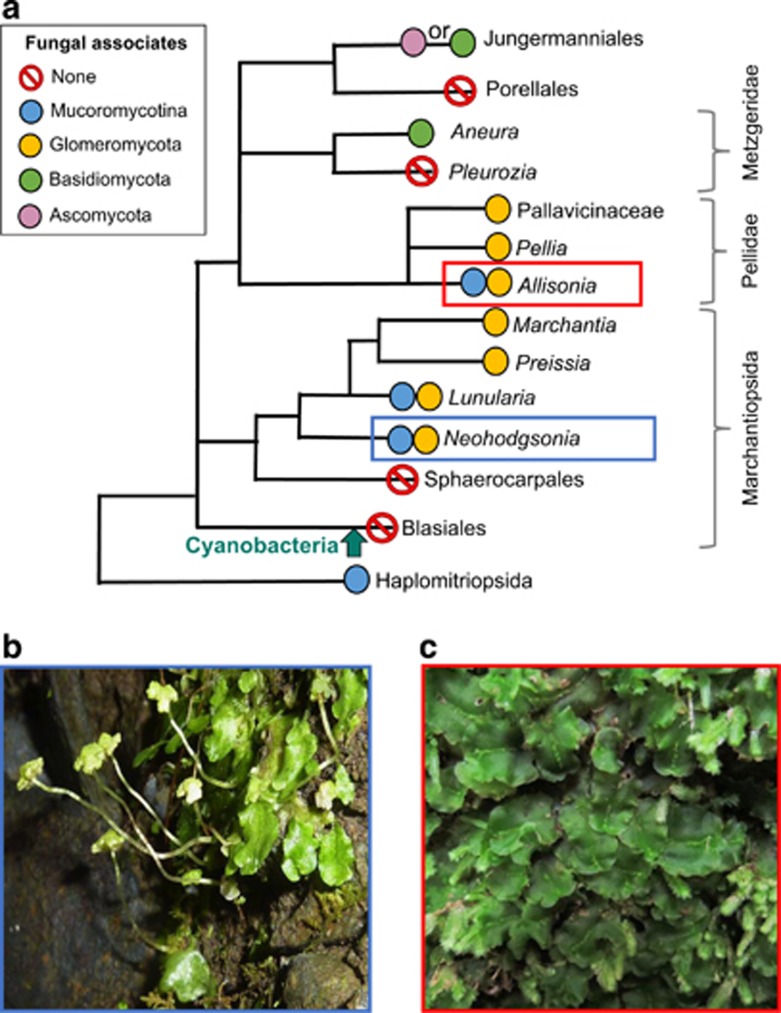
Liverwort phylogeny and species used in the present study. (**a**) Liverwort phylogeny (following Wikström *et al.*, 2009) showing key nodes alongside commonly associated fungal symbionts (James *et al.*, 2006; Pressel *et al.*, 2008; Bidartondo and Duckett, 2010; Humphreys *et al.*, 2010; Pressel *et al.*, 2010; Bidartondo *et al.*, 2011; Field *et al.*, 2012; Desirò *et al.*, 2013). Plants of (**b**) *Allisonia cockaynei* and (**c**) *Neohodgsonia mirabilis* photographed in the field (photo credits: KJ Field and JG Duckett).

Application of universal DNA primers, enabling detection of fungi beside Glomeromycota, together with detailed physiological and cytological observations, have now established that the earliest branching lineage of extant liverworts, the Haplomitriopsida (Heinrichs *et al.*, 2005, 2007; Crandall-Stotler *et al.*, 2009; Wikström *et al.*, 2009), often form mutualistic mycorrhiza-like associations exclusively with Mucoromycotina fungi (Bidartondo *et al.*, 2011; Field *et al.*, 2015a). This partially saprotrophic fungal lineage is basal or sister to the Glomeromycota (James *et al.*, 2006; Lin *et al.*, 2014), raising the hypothesis that plant–Mucoromycotina associations represent the ancestral mycorrhizal type for land plants and that these were replaced by the strictly biotrophic Glomeromycota as plants evolved and soil organic matter accumulated (Bidartondo *et al.*, 2011). Although some early branching clades of land plant taxa have been found to associate exclusively with one or other of these fungal groups, representatives of nearly all extant early branching clades of land plants examined thus far host both fungal lineages, sometimes simultaneously (Desirò *et al.*, 2013; Rimington *et al.*, 2015, and a recent report for *Haplomitrium mnioides* by Yamamoto *et al.*, 2015) (Figure 1a). These discoveries point to more versatile and shifting evolutionary scenarios in early plant–fungus symbioses than hitherto assumed (Field *et al.*, 2015b), suggesting that the ability to engage in simultaneous partnerships with both Mucoromycotina and Glomeromycota fungi may be a basal trait (Desirò *et al.*, 2013; Rimington *et al.*, 2015).

The latest evidence (Field *et al.*, 2015a) shows that liverwort–Mucoromycotina symbioses functionally differ from those between liverworts and Glomeromycota fungi (Field *et al.*, 2012) in their ability to maintain efficiency of carbon-for-nutrient exchange between partners across atmospheric CO_2_ concentrations (a[CO_2_]). The conditions in these studies simulate the 90% a[CO_2_] drop coincident with the diversification of terrestrial ecosystems through the Palaeozoic (Berner, 2006; Franks *et al.*, 2014). Although the symbiotic functional efficiency of liverwort–Glomeromycota associations was severely compromised by a simulated Palaeozoic fall in a[CO_2_], that of Haplomitriopsida liverwort–Mucoromycotina partnerships was unaffected or increased under the modern-day a[CO_2_] scenario. These findings parallel those in Glomeromycota-associated sporophytes of some vascular plants, which also increased in functional efficiency under lower a[CO_2_] (Field *et al.*, 2012). Therefore, the hypothesis that Mucoromycotina fungi, switching from saprotrophy to facultative biotrophy, facilitated the evolution and diversification of early land plants under a high a[CO_2_] and were among the first fungi to form mutualistic symbioses with plants is strengthened (Bidartondo *et al.*, 2011). It remains an open question as to why dual Mucoromycotina/Glomeromycota plant–fungus partnerships today are often restricted to early-branching lineages of land plants (Figure 1a) and thus ‘lost out' to Glomeromycota-specific ones in later-branching plant lineages, such as the angiosperms (Field *et al.*, 2015b).

We investigated the functionality and detailed cytology of the dual fungal associations in wild-collected *Neohodgsonia mirabilis*, the sister taxon to all other complex thalloid liverworts harbouring mycorrhiza-like associations, and *Allisonia cockaynei* in the earliest divergent clade of simple thalloid liverworts (Forrest *et al.*, 2006; Crandall-Stotler *et al.*, 2008, 2009; Villarreal *et al.*, 2015) (Figure 1). Using molecular methods, we found that both liverworts hosted simultaneous Glomeromycota and Mucoromycotina fungal partners (see Results). We used a combination of isotope tracers under a modern ambient a[CO_2_] of 440 p.p.m. and a simulated Palaeozoic (c. 410–390 Ma) atmosphere of 1500 p.p.m. [CO_2_] (Franks *et al.*, 2014).

We aimed to answer the following questions;

1. Is there an enhanced carbon cost to liverworts associated simultaneously with Mucoromycotina and Glomeromycota fungi compared with those harbouring single fungal symbionts?
2. Do plants with dual colonization by Mucoromycotina and Glomeromycota fungi benefit from enhanced nutrient gain in comparison to those harbouring single fungal associations?
3. Are the costs decreased and benefits increased by elevated a[CO_2_] for liverworts maintaining dual symbioses with both Mucoromycotina and Glomeromycota fungi?
4. Are there any characteristic cytological signatures of dual fungal symbiosis as opposed to single fungal associations?

## Materials and methods

### Plant material and growth conditions

The liverworts *Neohodgsonia mirabilis* (Perss.) Perss. and *Allisonia cockaynei* (Steph.) RM Schust. were collected from the South Island of New Zealand in April 2012, and vouchers were deposited in the Natural History Museum, London. We planted the liverworts directly into pots (120 mm diameter × 100 mm depth) soon after collection. Native soil surrounding liverwort rhizoids was left intact to act as a natural inoculum, and pots were carefully weeded regularly to remove any other plant species.

Based on the methods of Johnson *et al.* (2001), we inserted three mesh-windowed cylindrical cores (Supplementary Figure S1) into each experimental pot. The mesh covering the cores was fine enough to exclude liverwort rhizoids but allows the ingrowth of fungal hyphae. Two of the cores were filled with a homogeneous mixture of acid-washed silica sand (89% core volume), native soil gathered from around the rhizoids of wild plants (10% core volume) and fine-ground tertiary basalt (1% core volume) to act as fungal bait (Field *et al.*, 2012). The third core was filled with glass wool and enabled below-ground respiration sampling throughout the ^14^C-labelling period.

We maintained plants in controlled environment chambers (BDR16, Conviron, Winnipeg, MB, Canada) with settings chosen according to those of the plant's natural environment (see Supplementary Information). Each species was grown at either 440 p.p.m. a[CO_2_] (*n*=10) or at a simulated early-Palaeozoic a[CO_2_] concentration of 1500 p.p.m. (*n*=10) (Berner, 2006, Franks *et al.*, 2014). a[CO_2_] was monitored using CARBOCAP GMP343 CO_2_ sensors (Vaisala, Birmingham, UK) and maintained through addition of gaseous CO_2_. Cabinet settings and contents were alternated every 2 weeks, and we regularly rotated all pots within cabinets. Plants were acclimated to chamber/growth regimes for 12 weeks to allow establishment of mycelial networks within pots.

### Molecular identification of fungal associates

Wild *Neohodgsonia* and *Allisonia* thalli were prepared for molecular analysis within 1 day of collection and immediately following our isotope labelling experiments at the end of the growth period at different a[CO_2_]. We dissected both plant species in the same way to leave the central part of the thallus and rhizoidal ridge (2–3 mm^2^) where fungal colonization is the highest. The DNA extraction, amplification and sequencing were performed as per the methods of Gardes and Bruns (1993), Desirò *et al.* (2013) and Field *et al.* (2015a) (see Supplementary Information). Sequence identity was inferred from their most closely related BLAST hits (Altschul *et al.*, 1997). Bayesian inference was used to confirm the fungal identity of samples shown to be Glomeromycota or Mucoromycotina by BLAST. Sequences were aligned with reference DNA sequences from GenBank (Benson *et al.*, 2005) using MUSCLE alignment algorithms (Edgar, 2004) within MEGA v. 5.1 (Tamura *et al.*, 2011). We tested evolutionary models in MEGA and selected HKY85 (nst=2) with invgamma rates for Bayesian analysis using MrBayes (Huelsenbeck and Ronquist, 2001).

### Quantification of fluxes of C, ^33^P and ^15^N between liverworts and fungi

After the 12-week acclimation period, we introduced 100 μl of an aqueous mixture of ^33^P-labelled orthophosphate (specific activity 148 GBq mmol^−1^, total 111 ng ^33^P added) and ^15^N-ammonium chloride (1 mg ml^−1^) into one of the soil-filled mesh cores in each pot and 100 μl distilled water into the control core via the installed capillary tubes. Cores in which isotope tracers were introduced were left static in half of the pots to preserve direct hyphal connections with the liverworts. In the remaining half, labelled cores were rotated through 90°, severing the hyphal connections between the plants and core soil immediately prior to addition of isotopes and every other day thereafter (Supplementary Figure S2).

We sealed the top of all soil cores with lanolin and caps 21 days after addition of the isotope tracers. Glass wool-filled cores were sealed with a rubber septum (SubaSeal, Sigma). We then sealed each pot into a 3-l, gas-tight labelling chamber and added 2 ml 10% lactic acid to 15 μl Na^14^CO_3_ (specific activity 2.04 TBq mmol^−1^) in a cuvette within the chamber prior to illumination at 0700 hours. This resulted in the release of a 1.1-MBq pulse of ^14^CO_2_ gas. Pots were maintained under growth chamber conditions, and 1 ml of labelling-chamber headspace gas was sampled after 1 h and every 4 h thereafter. Below-ground gas was sampled via the glass-wool filled core after 1 h and every 2 h thereafter to monitor below-ground respiration and ^14^C flux for around 17 h (see Supplementary Information for further details).

### Plant harvest and sample analyses

Plant and soil materials were separated, freeze-dried, weighed and homogenized. In all, 10–30 mg of homogenized samples were digested in 1 ml of concentrated H_2_SO_4_. These were heated to 365 °C for 15 min, and 100 μl H_2_O_2_ was added to each sample when cool. Samples were reheated to 365 °C, and each clear digest solution was diluted to 10 ml with distilled water. Two ml of each diluted digest were then added to 10 ml of the scintillation cocktail Emulsify-safe (Perkin Elmer, Beaconsfield, UK) and quantified through liquid scintillation. ^33^P transferred to the plant via fungal mycelium was then calculated as detailed in Supplementary Information (Cameron *et al.*, 2007).

^15^N abundance was determined using Isotope Ratio Mass Spectrometry (IRMS). Between 2 and 5 mg of freeze-dried, homogenized plant tissue was weighed out into 6 × 4 mm^2^ tin capsules (Sercon Ltd, Crewe, UK) and analysed using a continuous flow IRMS (PDZ 2020 IRMS, Sercon Ltd). Air was used as the reference standard, and the IRMS detector was regularly calibrated to commercially available reference gases.

^14^C activity was quantified through sample oxidation and liquid scintillation. Approximately 10–100 mg of freeze-dried sample was placed in Combusto-cones (Perkin Elmer) before oxidation (Model 307 Packard Sample Oxidiser Isotech, Chesterfield, UK). CO_2_ released through oxidation was trapped in 10 ml Carbosorb prior to mixing with 10 ml Permafluor. Total carbon (^12^C+^14^C) fixed by the plant and transferred to the fungal network was calculated as a function of the total volume and CO_2_ content of the labelling chamber and the proportion of the supplied ^14^CO_2_ label fixed by the plants. The difference in carbon between the static and rotated cores is taken as equivalent to the total C transferred from plant to symbiotic fungus within the soil core, noting that a small proportion will be lost through soil microbial respiration. The total carbon budget for each experimental pot was calculated using equations from Cameron *et al.* (2006), which are detailed in Supplementary Information.

Data from *Allisonia* and *Neohodgsonia* are compared in the discussion to published and unpublished data from *Haplomitrium* and *Treubia* associated exclusively with Mucoromycotina fungi obtained from experiments using identical conditions within the same controlled environment growth chambers (see Field *et al.*, 2015a). Data are also presented alongside previously published data for *Preissia* and *Marchantia* associated only with Glomeromycota fungi from experiments using near-identical experimental conditions within the same controlled environment growth chambers (Field *et al.*, 2012). In these experiments, pots were filled with soil from dune slacks at Aberfraw, Anglesey, UK (Grid Reference: SH 397 648) but were otherwise identical to those of all our other experiments.

### Ultrastructural analyses

We processed plants that were wild-collected and from experiments where they were grown at two a[CO_2_] for transmission and scanning electron microscopy as described previously (Duckett *et al.*, 2006). For transmission electron microscopy, thalli were fixed in 3% glutaraldehyde, 1% fresh formaldehyde and 0.75% tannic acid in 0.05 m Na-cacodylate buffer, pH 7, for 3 h at room temperature. After rinses in 0.1 m buffer, samples were postfixed in buffered (0.1 m, pH 6.8) 1% osmium tetroxide overnight at 4 °C, dehydrated in an ethanol series and embedded in TAAB low viscosity resin via ethanol. Thin sections were cut with a diamond knife, stained with methanolic uranyl acetate for 15 min and in Reynolds' lead citrate for 10 min and observed with a Hitachi H-7100 transmission electron microscope (Hitachi High-Technologies Europe, Maidenhead, UK) at 100 kV. For scanning electron microscopy, we fixed thalli in 3% glutaraldehyde, dehydrated through an ethanol series, critical-point dried using CO_2_ as transfusion fluid, sputter coated with 390 nm palladium-gold and viewed them using a FEI Quanta scanning electron microscope (FEI, Hillsboro, OR, USA).

### Statistics

Effects of plant species, a[CO_2_] and the interaction between these factors on the C, ^33^P and ^15^N fluxes between plants and fungi from this and previous studies (Field *et al.*, 2012, 2015a) were tested using analysis of variance with additional *post-hoc* Tukey's tests where indicated. Data were checked for homogeneity of variance and normality. Where assumptions for analysis of variance were not met, data were transformed using log_10_ or arcsine-square-root as indicated in Table 1. Different letters in the figures denote statistical difference (*P*<0.05) in all the figures. All statistics were carried out using the statistical software package R 3.1.2 (R Core Team, 2012).

## Results

### Molecular identification of fungi

Molecular analyses of fungal partners (*n*=6) showed that *Allisonia* and *Neohodgsonia* plants freshly collected from the field and after our isotope tracing experiments are colonized by both Mucoromycotina and Glomeromycota fungi (Supplementary Figure S3). The Mucoromycotina fungi identified here were the same as those found previously in wild populations of both species (Bidartondo *et al.*, 2011) belonging to groups I and H (*sensu*, Desirò *et al.*, 2013) in *Neohodgsonia* and *Allisonia*, respectively. The Glomeromycota fungal associates were exclusively Glomerales in *Allisonia* while *Neohodgsonia* harboured members of both Glomerales and Archaeosporales. Sequences are deposited in GenBank (KR779272-KR7792784).

### Plant biomass

**Figure 2:**
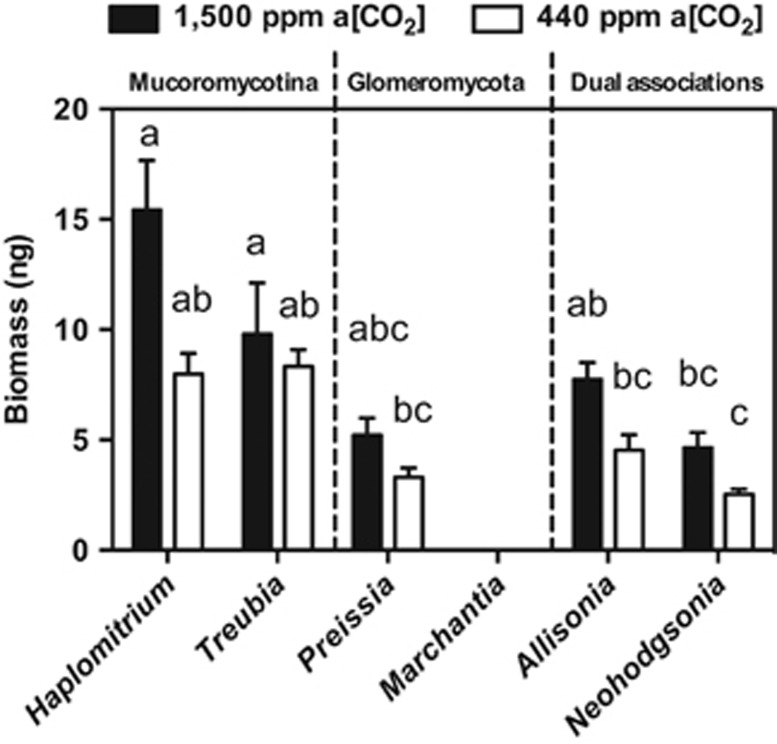
Mean total plant biomass (dry) at the end of experimental period in five liverwort species (Field *et al.*, 2012, 2015a) at both 1500 p.p.m. a[CO_2_] (black bars) and 440 p.p.m. a[CO_2_] (white bars). Error bars show s.e.m. (*n*=4 for all species); different letters denote statistical difference where *P*<0.05 (Tukey's *post hoc*).

Overall, there was a consistent trend of liverworts achieving greater biomass when grown at a[CO_2_] of 1500 p.p.m. compared with 440 p.p.m. a[CO_2_] (Figure 2). We found greater biomass of both *Allisonia* (41%) and *Neohodgsonia* (45%) grown at 1500 p.p.m. a[CO_2_] compared with those grown at a[CO_2_] of 440 p.p.m.

### Liverwort-to-fungus carbon transfer

**Figure 3:**
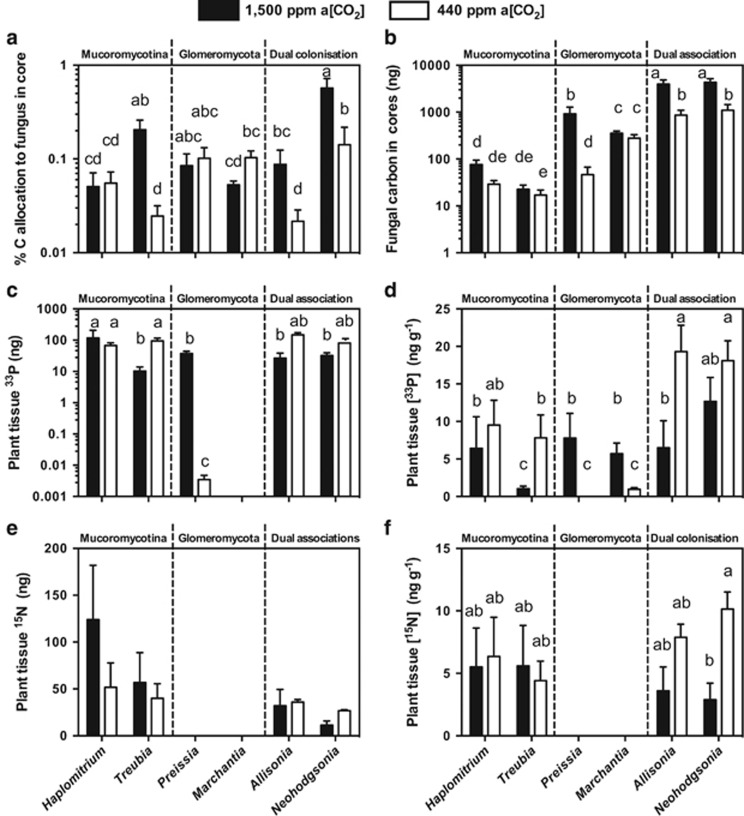
Carbon-for-nutrient exchange between liverworts and their fungal partners. (**a**) Percentage allocation of plant-derived carbon to fungi within soil cores, (**b**) total measured plant-fixed carbon transferred to fungi in soil for liverworts with different fungal associations (Mucoromycotina-only, Glomeromycota-only and dual fungal associations; Field *et al.*, 2012, 2015a); (**c**) total plant tissue ^33^P content (ng) and (**d**) tissue concentration (ng g^−1^) of fungal-acquired ^33^P in six liverwort species with different fungal associations under 1500 p.p.m. (black bars) a[CO_2_] and 440 p.p.m. (white bars) a[CO_2_] (Field *et al.*, 2012, 2015a); (**e**) total tissue ^15^N content (ng) and (**f**) concentration (ng g^−1^) of fungal-acquired ^15^N in four liverwort species with different fungal associations (Field *et al.*, 2015a) at both 1500 p.p.m. (black bars) and 440 p.p.m. (white bars) a[CO_2_]. In all panels, error bars show ±s.e.m. Different letters represent where *P*<0.05 (analysis of variance, Tukey's *post hoc*; see Table 1). In panels (**a**) and (**b**), *n*=6 for all species apart from *Marchantia*, where *n*=4; (**c**–**e**) *n*=4, where data are available.

Both *Allisonia* and *Neohodgsonia* allocated around four times more photosynthate to their fungal symbionts under the simulated Palaeozoic a[CO_2_] (Figure 3a) compared with the lower [CO_2_] (Figure 3a). In terms of total carbon transferred from plants to fungal partners (Figure 3b), each liverwort species transferred more carbon to their fungal symbionts at 1500 p.p.m. a[CO_2_] than at 440 p.p.m. a[CO_2_], this difference being significant in *Allisonia* and *Neohodgsonia*. As such, the dual fungal symbioses of *Neohodgsonia* and *Allisonia* have a greater total carbon ‘cost' at both a[CO_2_] than any of the other Glomeromycota– or Mucoromycotina–liverwort symbioses (Figure 3b).

### Fungal transfer of ^33^P and ^15^N to host liverworts

*Allisonia* and *Neohodgsonia* acquire 78% and 67% more ^33^P, respectively, at 440 p.p.m. compared with 1500 p.p.m. a[CO_2_], also reflected in plant tissue [^33^P] (Figures 3c and d). When grown at the 1500 p.p.m. a[CO_2_], the liverworts with dual fungal symbionts showed reduced total ^33^P uptake (Figure 3c), resulting in greatly reduced ^33^P concentrations in their tissues (Figure 3d).

The total uptake and assimilation of ^15^N is reduced by 11% in *Allisonia* and 57% in *Neohodgsonia* at 1500 p.p.m. a[CO_2_] compared with 440 p.p.m. a[CO_2_] (Figure 3e). In terms of tissue concentration, the same trend is amplified with [^15^N] being far greater by 250% and 119% in *Allisonia* and *Neohodgsonia*, respectively, at 440 p.p.m. a[CO_2_] compared with when plants are grown at 1500 p.p.m. a[CO_2_] (Figure 3f).

### Nutrient-for-carbon exchange efficiency

**Figure 4:**
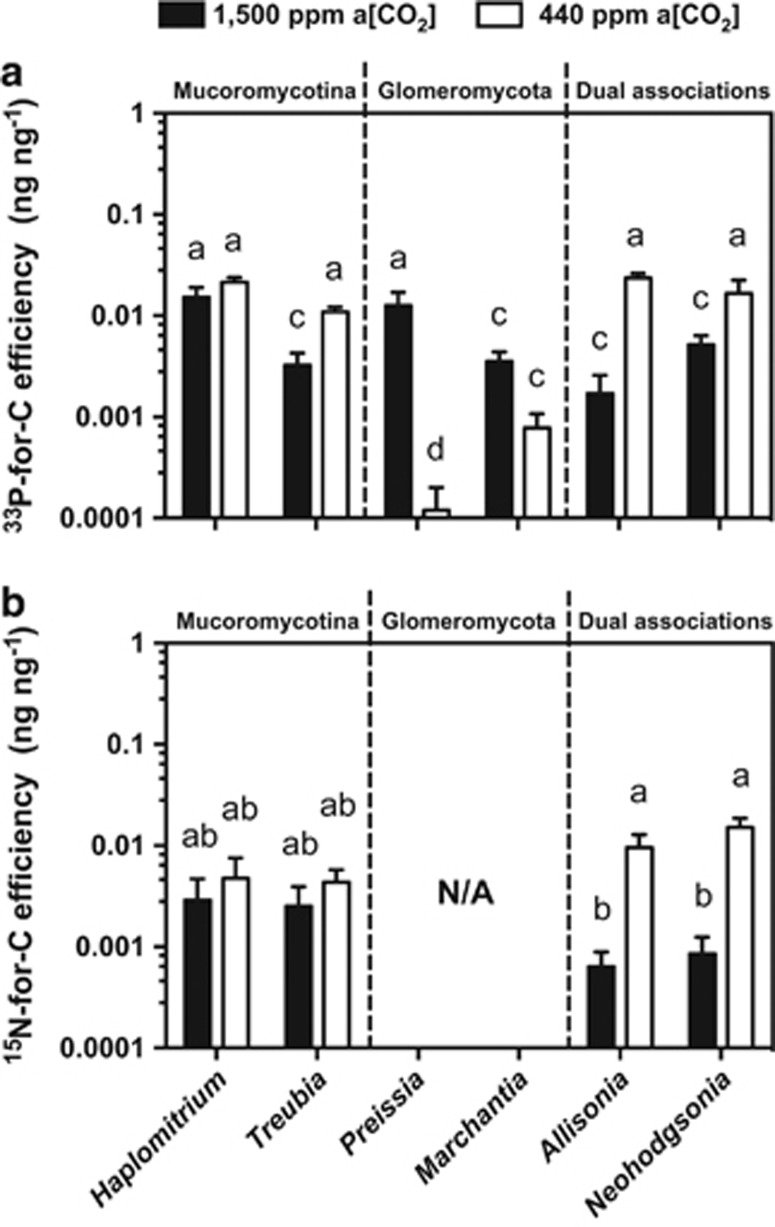
Nutrient-for-carbon exchange efficiencies between liverworts and their fungal partners. (**a**) ^33^P-for-carbon and (**b**) ^15^N-for-carbon efficiency for different liverwort species with different fungal associations under both 1500 p.p.m. (black bars) and 440 p.p.m. (white bars) a[CO_2_] (Field *et al.*, 2012, 2015a,b). Error bars show s.e.m. (*n*=4 for all species). Different letters indicate where *P*<0.05 (analysis of variance, Tukey's *post hoc*).

^33^P-for-C exchange efficiency in *Allisonia* was >13 times greater at 440 p.p.m. a[CO_2_] than it was at 1500 p.p.m. a[CO_2_] (Figure 4a). The same pattern was true in *Neohodgsonia*, with three times greater ^33^P-for-C exchange efficiency at the lower a[CO_2_] (Figure 4a). ^15^N-for-C exchange was an order of magnitude greater in both *Allisonia* and *Neohodgsonia* at the lower a[CO_2_] compared with the elevated a[CO_2_] (Figure 4b).

### Cytology of colonization

The cytology of dual colonization by Mucoromycotina and Glomeromycota fungi in wild plants of *Neohodgsonia* and *Allisonia* is described here for the first time. As our detailed electron microscopic analyses revealed no major differences, only a minor one in *Allisonia* (detailed below), between wild and experimental plants grown at contrasting a[CO_2_] (440 and 1500 p.p.m.), the results are presented together unless otherwise stated.

### Neohodgsonia mirabilis

**Figure 5:**
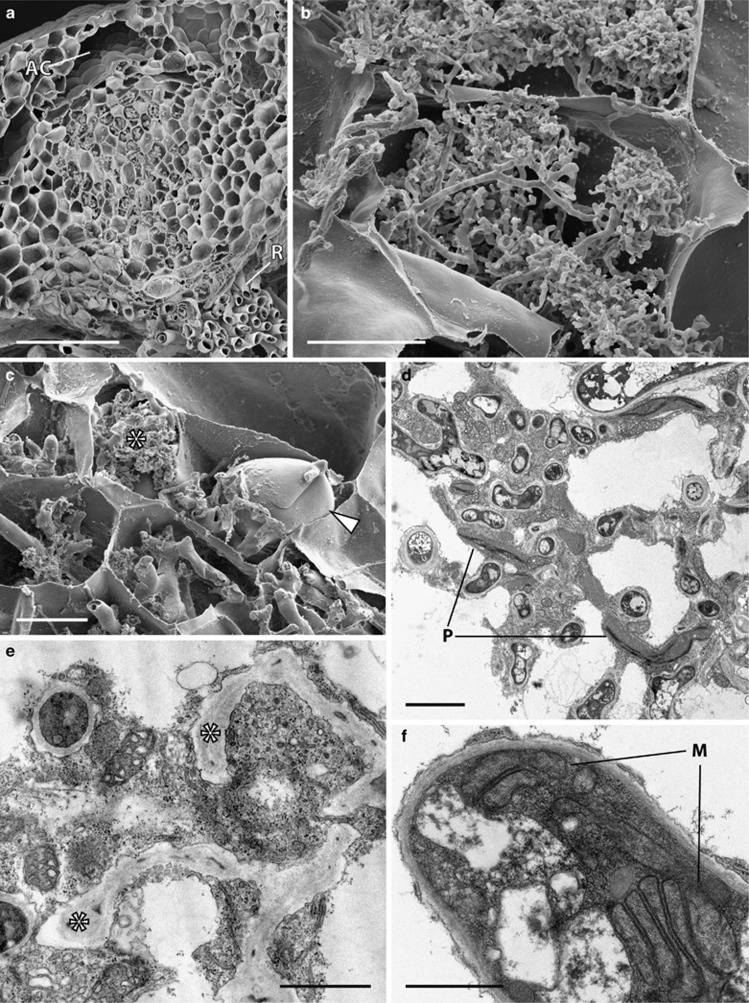
Cytology of *Neohodgsonia mirabilis* grown at 440 and 1500 p.p.m. a[CO_2_]. Scanning (**a**–**c**) and transmission (**d**–**f**) electron micrographs (TEM). Both the distribution and cytology of the association remained the same between a[CO_2_] treatments and are illustrated here in plants grown at 440 p.p.m. a[CO_2_]. (**a**) Fungal colonization zone extending from the rhizoid (R) bearing ventral surface of the thallus to just below the dorsal air chambers (AC). (**b**, **c**) Young arbuscules (**b**) and collapsed ones (**c**) (*) adjacent to a large vesicle (arrowed). (**d**) fungal hyphae surrounded by active host cytoplasm. Note the plastids (P) with well-developed thylakoid systems but largely devoid of starch. (**e**) Degenerated arbuscular hyphae (*) surrounded by healthy host cytoplasm. (**f**) Fungal hyphae typically contain multiple mitochondrial stacks (M). Scale bars: (**a**) 200 μm; (**c**) 50 μm; (**b**) 20 μm; (**d**) 3 μm; (**e**, **f**) 1 μm.

Fungal colonization occupies the central thallus midrib, extending all the way from the rhizoid-bearing ventral surface, the point of fungal entry (see Supplementary Information), to just below the large dorsal air chambers (Figure 5a). Fungal structures comprise numerous arbuscules at various stages of development, from young (Figure 5b) to collapsed and large vesicles occupying a significant proportion of the host cell (Figure 5c). Healthy (Figure 5d) and degenerated arbuscules (Figure 5e), large living hyphae, vesicles and active host cytoplasm are most often present in the same host cell. Fungal trunk hyphae and arbuscular hyphae are surrounded by the host plasma membrane, and the cytoplasm of the host cells comprises numerous Golgi bodies, mitochondria, plastids and microbodies (Figures 5d and e). The latter have a well-developed thylakoid system but are largely devoid of starch deposits (Figure 5d). Fungal hyphae are aseptate and often contain multiple mitochondrial stacks, each comprising of 5–6 mitochondria (Figure 5f).

### Allisonia cockaynei

**Figure 6:**
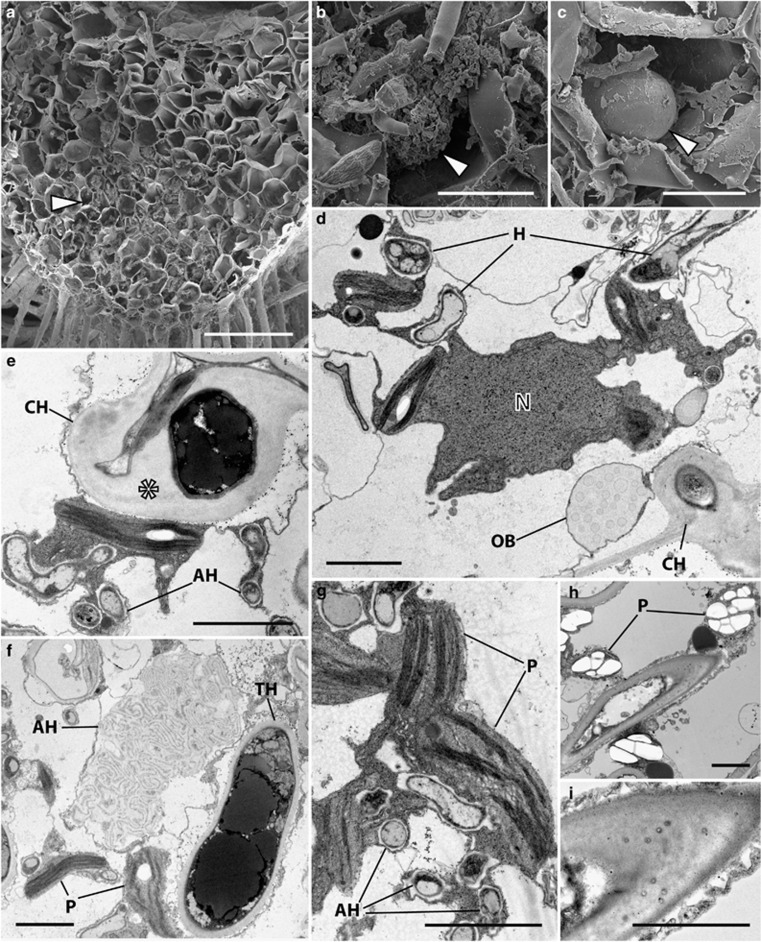
Cytology of *Allisonia cockaynei* grown at 440 and 1500 p.p.m. a[CO_2_]. Scanning (**a**–**c**) and transmission (**e**–**i**) electron micrographs (TEM). There was no change in the overall distribution of fungal colonization and in the cytology of the fungus between [CO_2_] treatments, both illustrated here in plants grown at 440 p.p.m. a[CO_2_] except for panels (**h** and **i**). (**a**) Fungal colonization zone (arrowed) occupying the first 10 cell layers from the rhizoid-bearing ventral surface. (**b**) Collapsed arbuscules (arrowed) and (**c**) large vesicle (arrowed). (**d**) Host cell with active cytoplasm in close association with fungal hyphae (H). Note the colonizing hypha (CH) traversing the host cell wall. N, nucleus; OB, oil body. (**e**) colonizing hypha with thick layer of fibrillar material (*) in between the fungus cell wall and the host plasma membrane (arrowed) and thin-walled arbuscular hyphae (AH) in close proximity to plastids (P). (**g**) Arbuscular hyphae in close association with starch-free plastids. (**h**) In plants grown at 1500 p.p.m. a[CO_2_] plastids have prominent starch deposits. (**i**) Plasmodesmata-like channels are present in the fibrillar material that surrounds the colonizing hyphae. Scale bars: (**a**) 200 μm; (**b**, **c**) 20 μm; (**d**–**i**) 3 μm.

Fungal entry is via the rhizoids (Supplementary Figure S4) with the fungal zone occupying the central region of the thallus, generally the first 10 cell layers from the rhizoid-bearing ventral side with approximately 1/3 of the thallus midrib remaining free of fungal structures (Figure 6a). These comprise large hyphae, arbuscules (Figure 6b) and prominent vesicles (Figure 6c). Host cells are characterised by active cytoplasm, including numerous mitochondria and plastids in close association with the fungus (Figure 6d). Colonizing hyphae traverse the walls of adjacent host cells and have a thick layer of fibrillar material in between the fungus cell wall and the host plasma membrane that surrounds them (Figure 6d) while arbuscular hyphae are characterized by thin cell walls (Figure 6e). These are often collapsed while the colonizing hyphae and host cytoplasm surrounding them persist (Figure 6f). Whereas the plastids of wild plants and those grown at 440 p.p.m. a[CO_2_] contain little or no starch deposits (Figure 6g), those of plants grown at 1500 p.p.m. a[CO_2_] have prominent starch grains (Figure 6h). The large colonizing hyphae of wild and experimental plants grown under contrasting a[CO_2_] regimes are all characterized by plasmodesmata-like channels in the fibrillar material that surrounds them (Figure 6i).

## Discussion

The currently emerging paradigm considers the Mucoromycotina symbiosis with plants to have evolved prior to the emergence of plant–Glomeromycota fungal symbioses (Bidartondo *et al.*, 2011). Moreover, until very recently it has been assumed that early diverging lineages of plants associate with only Glomeromycota (Wang and Qui, 2006). In direct contrast to this, our work shows that basal liverwort lineages (Figure 1a) form simultaneous mutualistic symbioses with both Mucoromycotina and Glomeromycota fungi. This raises novel questions regarding mycorrhizal evolution; given the global radiation and dominance of glomeromycotean symbioses, why have associations with Mucoromycotina fungi persisted? We can now begin to answer this question with the present demonstration that dual associations are significantly more efficient at modern day atmospheric CO_2_ compared with Palaeozoic CO_2_, whereas single fungal group partnerships are either unaffected by a[CO_2_] (Mucoromycotina fungi) or are less efficient under modern day a[CO_2_] (Glomeromycota fungi). This trade-off provides a physiological niche facilitating the persistence of plant symbioses with Mucoromycotina fungi, singly and in dual partnerships with Glomeromycota fungi to the present day.

### Physiological costs and benefits

Our experiments reveal that *Neohodgsonia* and *Allisonia* with dual Glomeromycota and Mucoromycotina fungal associations allocated greater percentages and total amounts of photosynthate to their fungal partners at 1500 p.p.m. a[CO_2_] than at 440 p.p.m. a[CO_2_] (Figures 3a and b). Our previous studies show that in terms of percentage of carbon allocation, Mucoromycotina partners of *Treubia* receive seven times greater percentage allocation of plant-fixed carbon at 1500 p.p.m. a[CO_2_] compared with at 440 p.p.m. a[CO_2_]. There is little difference in percentage of C allocation in *Haplomitrium* while in *Marchantia* and *Preissia* the percentage of C allocation to Glomeromycota fungi is 1.9 and 1.2 times greater, respectively. This likely resulted in the greater biomasses recorded in all liverworts at elevated a[CO_2_] (Figure 2).

In all of the combinations of liverwort–fungal symbioses examined thus far, partnerships in which there is a Mucoromycotina fungal symbiont (that is, in *Haplomitrium*, *Treubia*, *Allisonia* and *Neohodgsonia*) display increased ^33^P-for-C and ^15^N-for-C exchange efficiency at 440 p.p.m. a[CO_2_] compared with at 1500 p.p.m. a[CO_2_] (Figure 4). In liverwort–Glomeromycota symbioses, the opposite trend is apparent, with ^33^P-for-C being several orders of magnitude lower in both *Marchantia* and *Preissia* at 440 p.p.m. compared with at 1500 p.p.m. a[CO_2_] (Figure 4a).

Decreased fungal-acquired nutrient uptake in liverworts with Mucoromycotina fungal partners (either single or dual colonizations) at elevated a[CO_2_] seems at first counter-intuitive, particularly given their larger biomass (Figure 2) and increased photosynthate allocation to fungal partners (Figures 3a and b) in those conditions. However, it is possible that the plants in our experimental pots experienced nutrient limitation (for P, N or both). This seems likely considering the lack of plant-available nutrients in the surrounding sand and its limited accessibility within the soil cores. As such, when a[CO_2_] is at 1500 p.p.m., the liverworts likely produced excess photosynthates that they might have been unable to utilize for growth or reproduction owing to nutrient limitation. As liverworts are structurally simple plants, with no vasculature or specialized storage organs to provide transport and storage of excess carbohydrates (Kenrick and Crane, 1997), surplus sugars must be either stored as insoluble starch granules within the thallus (observed here in *Allisonia;* Figure 6h), supplied directly to fungal partner(s) (see Figures 3a and b), or be released into the surrounding soil as exudates.

It is likely that the greater C allocation we observed from liverworts to Mucoromycotina fungal partners in our experiments allows increased hyphal proliferation and fungal sporulation. Given that these processes are demanding in terms of energy and resources (Denison and Kiers, 2011), the fungus would have greater N and P requirements and therefore may assimilate more of the nutrients acquired from its surroundings, rather than surrender them in return for plant carbohydrate. This may provide a mechanism to explain our observations of reduced fungal-acquired nutrient uptake in Mucoromycotina-exclusive and dual Mucoromycotina/Glomeromycota-partnered liverworts at elevated a[CO_2_], even with enhanced C allocation to fungal partners (Figures 4 and 5). It is also possible that there are further non-nutritional benefits for liverworts in symbiosis with Mucoromycotina fungi that have not been explored here, such as enhanced disease and/or herbivore resistance (Cameron *et al.*, 2013).

In contrast, the liverworts partnered exclusively with obligately biotrophic Glomeromycota fungi in previous experiments (that is, *Marchantia* and *Preissia* in Field *et al.*, 2012) operated a more linear exchange of nutrients-for carbon. In this scenario, more photosynthate is supplied to the fungal mycelium at elevated a[CO_2_], which in turn supplies more ^33^P to the host plant. At 440 p.p.m. a[CO_2_], the plant does not maintain the same supply of photosynthates to the fungus, and so the fungus does not return as much nutrient to its host. This pattern of ‘tit-for-tat' reciprocity in plant–Glomeromycota symbiosis has previously been demonstrated in various vascular plant species, both in root-organ culture systems (Kiers *et al.,* 2011) and in whole-plant experiments (Hammer *et al.*, 2011; Fellbaum *et al.*, 2014). Here we demonstrate that this model does not apply in our case of a plant symbioses involving more than one fungal partner and involving Mucoromycotina fungi.

It is possible that by allocating excess photosynthates directly to Mucoromycotina fungal partners, rather than releasing them as C-rich plant exudates, the liverworts avoid providing excess carbohydrate resources to surrounding saprotrophic microbes. This may help to reduce nutrient immobilization by free-living saprotrophic microorganisms and damage or toxicity caused by potential microbial pathogens (Otten *et al.*, 2004). These potential benefits to the plants may contribute to the maintenance of Mucoromycotina fungal partnerships even in plants that can form symbiotic associations with Glomeromycota fungi and may explain why these have not been lost entirely from extant plants through evolutionary time (Rimington *et al.*, 2015). If excess photosynthates are released as exudates from the liverworts, they are likely to enhance nutrient immobilization and increase their nutrient limitation.

### Cytological characteristics

Our investigation reveals that the cytology of fungal colonization in both *Neohodgsonia* and *Allisonia* is typical of mycorrhizal associations involving Glomeromycota fungi; in both it comprises prominent vesicles and well-developed, short-lived arbuscules and/or fine hyphae surrounded by active host cytoplasm. The last feature is also typical of the intracellular phase in Mucoromycotina associations (Desirò *et al.*, 2013; Strullu-Derrien *et al.*, 2014; Rimington *et al.*, 2015; Field *et al.*, 2015a). However, the key feature of intracellular colonization in Haplomitriopsida–Mucoromycotina symbiosis—hyphal coils with terminal swellings (‘lumps') (Carafa *et al.*, 2003; Duckett *et al.*, 2006)—seems to be unique and has not been observed in any other liverwort–fungus partnerships, including those in *Neohodgsonia* and *Allisonia*.

Another diagnostic feature of Mucoromycotina colonization, intercellular fungal proliferation with the production of thick-walled spores in mucilaginous spaces, does occur across plant lineages, including hornworts and lycopods, but neither *Neohodgsonia* nor *Allisonia* develop mucilage-filled schizogenous intercellular spaces in their thalli. It is unsurprising therefore that in these two species we did not observe any of the major cytological differences between ambient and elevated a[CO_2_]-grown plants reported in the Haplomitriopsida (Field *et al.*, 2015a) as the latter were exclusively associated with the intercellular phase of fungal colonization. The single minor cytological difference observed between wild and experimental plants grown at contrasting a[CO_2_], and restricted to *Allisonia*, was the presence of far more starch granules within thalli of this species when grown at high a[CO_2_] (Figure 6h).

The only cytological features that may potentially be indicative of fungal identity in these dual symbioses are the fine/arbuscular and trunk/colonizing hyphal diameters (Strullu-Derrien *et al.*, 2014). In Mucoromycotina–liverwort symbiosis, the fine hyphae range from 0.5 to 1.0 μm and the larger classes are 3–4 μm (*Haplomitrium*, *Treubia*), but in Glomeromycota–liverwort symbiosis (*Marchantia*, *Preissia*, *Pellia*) the corresponding dimensions are from 1 to 3 μm and from 4 to 8 μm. Measurements of *Neohodgsonia* and *Allisonia* reveal that the fine hyphae range from 0.6 to 1.2 μm, that is, mostly in the Mucoromycotina range, whereas the trunk hyphae, ranging from 3 to 8 μm, are more typical of Glomeromycota. In contrast, vesicles are consistently diagnostic of Glomeromycota. Thus, although the identification of the two different fungi in *Neohodgsonia* and *Allisonia* largely rests with the molecular evidence, there are indications from cytology for the presence of both Mucoromycotina and Glomeromycota fungi that could be further explored by cytochemical and cytogenetic techniques. Consequently, regarding the large number of previous studies, particularly in early-diverging plant lineages, in which electron microscopy has been used to describe mycorrhizal associations as ‘glomeromycotean', our findings suggest that cryptic Mucoromycotina associations may sometimes also be occurring simultaneously.

*In vitro* isolation and resynthesis experiments with liverworts known to engage in dual symbioses and whereby either of the two mycobionts is reintroduced in the host plant will help to determine cytological similarities and/or differences between the two fungal symbionts. Fluorescence *in situ* hybridization may allow localization of Glomeromycota and Mucoromycotina fungi co-existing in the same host plant to establish which structures belong to which fungus. In the meantime, it is essential that fungal identification is carried out using appropriately inclusive molecular techniques in any mycorrhizal or mycorrhizal-like symbiosis.

### Wider perspectives

Our findings indicate that under a modern near-ambient a[CO_2_], liverworts in partnership with Mucoromycotina, either in single or dual associations alongside Glomeromycota fungi, benefit from greater nutrient gain for carbon outlay than liverworts that maintain mutualistic symbioses with only a Glomeromycota fungal symbiont. From an evolutionary perspective, the relative increases in nutrient exchange efficiency of plants harbouring both types of symbiont at lower a[CO_2_] may at least partially explain why declining atmospheric a[CO_2_] over the course of the Palaeozoic would have favoured the retention of both functional types of symbiosis. However, it is important to note that plants living in 1500 p.p.m. a[CO_2_] were likely to experience other abiotic factors that changed as plants evolved, including soil mineralogy and nutrient supply.

The question remains whether Mucoromycotina fungal symbioses resemble an ancestral condition that gave way to dual (for example, *Neohodgsonia* and *Allisonia*) and then solely Glomeromycota symbiosis (for example, *Marchantia*, *Preissia*, *Conocephalum*) or whether co-evolution of plant and fungal symbioses have been more dynamic than previously thought (Field *et al.*, 2015b). Indeed, the liverwort phylogeny (Figure 1a) is associated with repeated losses and re-acquisitions of the same or different fungal symbionts. That liverwort clades supporting dual fungal partnerships have fungus-free sister groups, for example, the Sphaerocarpales and Blasiales (Pressel *et al.*, 2010), points to shifting fungal associations during liverwort evolution. Exclusive plant–Mucoromycotina fungal symbiosis being a basal trait is only supported by these associations being present in liverworts of the Haplomitriopsida (Bidartondo *et al.*, 2011), the sister group to all other liverworts (Forrest *et al.*, 2006; Crandall-Stotler *et al.*, 2008, 2009), with liverworts themselves being the earliest diverging land plant lineage (Alaba *et al.*, 2014; Cox *et al.*, 2014; Qiu *et al.*, 1998, 2006, 2007).

Mounting evidence that a large proportion of taxa in all extant early-diverging plant lineages (Desirò *et al.*, 2013; Rimington *et al.*, 2015; Field *et al.*, 2015a), and likely some Rhynie Chert fossil plants (Strullu-Derrien *et al.*, 2014), form dual symbiosis with both Mucoromycotina and Glomeromycota fungi now corroborates these simultaneous fungal partnerships as being an extremely ancient condition, coincident with the early evolution of land plants. Why some Haplomitriopsida liverworts do not engage in symbiosis with the ubiquitous Glomeromycota fungi remains enigmatic given the clear advantages of dual partnerships demonstrated here. Even less comprehensible are the obligate Glomeromycota relationships in thalloid liverworts such as *Marchantia* and *Pressia*, given that recent functional studies clearly demonstrated that the symbiotic functional efficiency of these partnerships is severely compromised by the fall in a[CO_2_] that occurred through land plant diversification (Field *et al.*, 2012). In this context, it is interesting to note that liverwort clades harbouring exclusively Glomeromycota fungi have much later divergence times than those able to associate with both fungal symbionts (Cooper *et al.*, 2012; Feldberg *et al.*, 2013). *Marchantia*, *Conocephalum* and *Preissia* most likely diverged during the Cretaceous (Wikström *et al.*, 2009; Villarreal *et al.*, 2015), a period of rapid angiosperm and polypodiaceous fern radiation (Schneider *et al.*, 2004). We hypothesize that during this period major changes in abiotic and biotic dynamics, both below ground and above-ground, led to the predominance of the biotrophic Glomeromycota fungi in land plant–fungal interactions. It is possible therefore that these Glomeromycota-specific liverworts evolved in Glomeromycota-dominated environments and never engaged in associations with Mucoromycotina fungi.

In this first assessment of the functionality and cytology of the dual symbiosis of plants with Mucoromycotina and Glomeromycota fungi, we were not able to distinguish between fungal partners using microscopical techniques nor relative carbon allocation to each fungal symbiont. Future research using axenic cultures of plants and symbiotic fungi may enable such comparisons to be made and is an area for future development. More targeted cytological techniques, such as fluorescence *in situ* hybridization, may provide further novel insights into the associations and should be pursued in the future. With the discovery of dual Mucoromycotina-Glomeromycota symbioses in early branching lineages of living vascular plants (Rimington *et al.*, 2015), it is now critical that we explore how far these might extend into seed plants.

## Figures and Tables

**Table 1:** Summary of differences in mycorrhizal functionality (*F* ratio from ANOVA) between *Neohodgsonia, Alisonia, Haplomitrium, Treubia, Preissia and Marchantia* at elevated a[CO_2_] (1500 p.p.m.) and ambient a[CO_2_] (440 p.p.m.)

	*df*	*Plant species*	*CO*_*2*_ *treatment*	*Species × CO*_*2*_
Biomass (g)	1, 30	16.276***	18.911***	1.937
Fungal carbon in cores (ng)^a^	1, 54	14.042***	31.334***	5.087***
Percentage of carbon allocation^b^	1, 54	5.756***	13.900***	3.278*
Total ^33^P uptake (ng)^b^	1, 30	4.498**	5.714*	6.483***
[^33^P] in plant tissue (ng g^−1^)	1, 36	6.259***	3.142	3.857*
Total ^15^N uptake (ng)	1, 20	1.889	0.953	0.822
[^15^N] in plant tissue (ng g^−1^)^a^	1, 20	0.235	1.147	1.304
^33^P-for-C efficiency (ng ng^−1^)	1, 36	46.220***	0.885	31.747***
^15^N-for-C efficiency (ng ng^−1^)	1, 20	0.413	13.523**	1.913

Abbreviations: ANOVA, analysis of variance; p.p.m., parts per million. *P<0.05, **P<0.01, ***P<0.001; *post-hoc* Tukey's test.

a Data have been log_10_ transformed to meet the assumptions for ANOVA.

b Data have been arcsine-square-root transformed to meet the assumptions for ANOVA.
