# Supplementary material for: Functional analysis of liverworts in dual symbiosis with Glomeromycota and Mucoromycotina fungi under a simulated Palaeozoic CO2 decline
Source: ISME J. 2015 Nov 27;10(6):1514–26. doi: 10.1038/ismej.2015.204 (PMC5029179; doi:10.1038/ismej.2015.204)
Supplement: Supplementary Information [file ismej2015204x1.pdf]

Functioning of dual Glomeromycota and Mucoromycotina fungal associations in liverworts with Palaeozoic changes in CO_2

Katie J. Field, William R. Rimington, Martin I. Bidartondo, Kate E. Allinson, David J. Beerling, Duncan D. Cameron, Jeffrey G. Duckett, Jonathan R. Leake and Silvia Pressel

Supplementary Information

Summary

This file provides specific details on methods referred to in the main text of the above article, including watering regime, mesh-covered core construction, published equations for calculation of carbon and nutrient fluxes between symbionts and details on molecular methods for fungal identification. Figure S1 and S2 provide an illustrations of the experimental systems, S3 depicts a phylogenetic tree showing the placement of mutualistic fungi colonising *Allisonia cockaynii* and *Neohodgsonia mirabilis* and S4 shows fungal entry in *Neohodgsonia mirabilis.*

Materials and Methods

Plant material

The pots were filled to 95% pot volume acid-washed silica sand and 5% Irish *Sphagnum* moss peat to aid water retention properties of the substrate.

Artificial rainwater (Olsson &Tyler, 1993) solution was prepared as follows:

	**mg l^{-1}**	**In 10 l (g)**
NH_4NO_3	5.6	0.058
NaCl	4.3	0.043
$MgSO_4.7H_2O$	2.1	0.023
H_2SO_4 (µl l^{-1})	30	300 µl
$NaH_2PO_4.2H_2O$	0.47	0.0047

K_2SO_4	1.2	0.012
CaCl	4.2	0.042

Rainwater solution was applied to plants within experimental systems as a fine mist every two days. Plants were misted until the surface of the plant was visibly wet.

Growth conditions within controlled environment growth chambers were set as follows: half light-saturating conditions for non-vascular plants of 50 µmol m^{-2} s^{-1} irradiance (Nobel, 1991; Fletcher *et al.,* 2006), 70% relative humidity, 12 h, 15°C day, 12°C night.

Construction of mesh-covered cores

Based on the methods of Johnson *et al.* (2001), each core had two windows cut into the below-ground portion (20 mm x 50 mm, see Fig. S1). The windows and base were covered by 10 µm pore size nylon mesh and sealed with a fast-setting cement (Polypipe Building Products, UK). This mesh size is fine enough to exclude liverwort rhizoids but allows the ingrowth of fungal hyphae. We perforated a fine-bore capillary tube using a needle, and installed it to run the full length of each of the cores (100 mm in length, 1.02 mm internal diameter; Portex, UK). The tubing ensured isotope was introduced and distributed evenly throughout the core volume. We sealed the capillary tube using fast-setting cement 5 mm from the bottom of the core in order to prevent excess isotope leaching from the bottom of the core.

Molecular identification of fungal associates

Wild *Neohodgsonia* and *Allisonia* were prepared for molecular analysis within one day of collection. We dissected both plant species in the same way to leave the central part of the thallus and rhizoidal ridge (2-3 mm^2) where fungal colonisation is highest. We performed two independent DNA extractions per plant using the method of Gardes & Bruns (1993) in combination with the QBioGene Gene-Clean kit. Genomic DNA was amplified using the 18S universal fungal primer set NS1 (White *et al*., 1990) and EF3 (Smit *et al*., 1999) which amplifies all known lineages of Mucoromycotina and Glomeromycota (Desirò *et al*., 2013). The following PCR settings were used in conjunction with Sigma JumpStart: 94 °C for 2 minutes, 34 cycles of 94 °C for 30 seconds, 53 °C for 30 seconds and 72 °C for 1 minute 30 seconds, followed by a final 72 °C for 7 minutes. We cloned the PCR products (TOPO TA,

Invitrogen, USA) and sequenced between four and eight colonies per DNA extraction. The PCR products of each colony were purified using Affymetrix ExoSAP-IT and sequenced using Big Dye v3.1 on an Applied Biosystems 3730 genetic analyser. The DNA was sequenced using the primers NS1, NS3 and NS5 (White *et al.,* 1990) which were edited and assembled into contigs (ca. 1,600 bp) using Geneious v5.6.4 (Biomatters, NZ). An identical method was used for the sequencing of the plants used in the isotope labelling experiments.

Plant harvest and sample analyses

Upon detection of maximal belowground ^{14}C flux following release of $^{14}CO_2$, 2ml of 2 M KOH was introduced to each chamber to trap residual $^{14}CO_{2(g)}$ in the chamber headspace. Soil cores were removed from pots and incubated in gas-tight containers with a further 2 ml 2 M KOH to trap $^{14}CO_2$ emitted through respiration. Soil cores were transferred to fresh containers and KOH every two hours for a further four hours. One ml of each 'used' KOH trap was transferred to vials containing 10 ml of the scintillation cocktail Ultima Gold (Perkin Elmer, Beaconsfield, UK) and the radioactivity of each sample determined through liquid scintillation. These data were used to calculate carbon budgets for each experimental pot (see SI for equations).

Plant and soil materials were separated, freeze-dried, weighed and homogenised using a Yellowline A10 Analytical Grinder (IKA, Germany).

^{33}P transfer from fungus to plant

The ^{33}P transferred from fungus to plant was determined using published equations (Cameron *et al.*, 2007):

$$M^{33}\mathrm{P} = \left\{\left[\frac{c\mathrm{DPM}/60}{S\mathrm{Act}}\right] M\mathrm{wt}\right\} Df \qquad (1)$$

Where M^{33}P = Mass of ^{33}P (mg), *c*DPM = counts as disintegrations per minute, *S*Act = specific activity of the source (Bq mmol^{-1}), *Df* = dilution factor and *M*wt = molecular mass of P.

Carbon transfer from plant to fungus

The difference in carbon between the static and rotated cores is equivalent to the total C transferred from plant to symbiotic fungus within the soil core. Total carbon assimilated by the plant was calculated using equation 2 (Cameron *et al.*, 2006):

$$T_{pf} = \left(\left(\frac{A}{A_{sp}}\right) m_a\right) + (P_r \times m_c)$$

(2)

Where T_{pf} = Transfer of carbon from plant to fungus, A = radioactivity of the tissue sample (Bq); A_{sp} = specific activity of the source (Bq Mol^{-1}), m_a = atomic mass of ^{14}C, P_r = proportion of the total ^{14}C label supplied present in the tissue; m_c = mass of C in the CO_2 present in the labelling chamber (g) (from the ideal gas law; Eqn. 3):

$$m_{cd} = M_{cd}\left(\frac{PV_{cd}}{RT}\right) \therefore m_c = m_{cd} \times 0.27292 \quad (3)$$

where m_{cd} = mass of CO_2 (g), M_{cd} = molecular mass of CO_2 (44.01 g mol^{-1}) P = total pressure (kPa); V_{cd} = volume of CO_2 in the chamber (0.003 m^3); R = universal gas constant (J K^{-1} mol^{-1}); T, absolute temperature (K); m_c, mass of C in the CO_2 present in the labelling chamber (g), where 0.27292 is the proportion of C in CO_2 on a mass fraction basis (Cameron *et al.*, 2008).

Supplementary figures

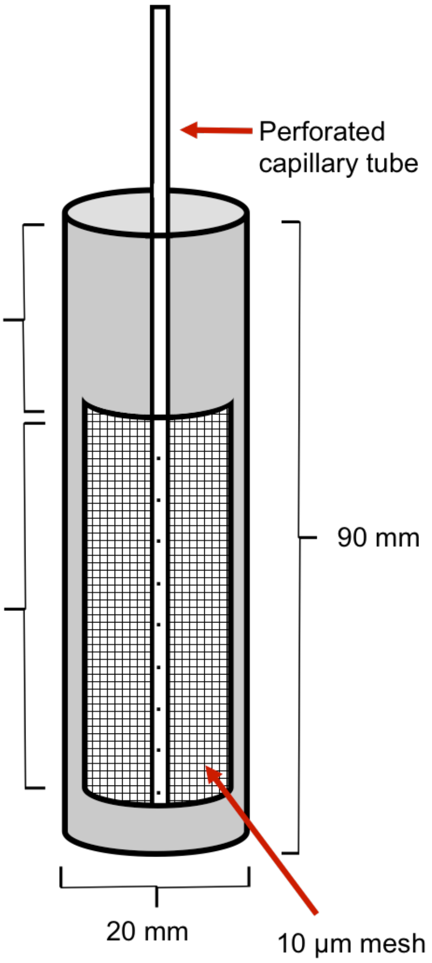

Figure S1. Schematic diagram of mesh-covered core (not drawn to scale).

]

A

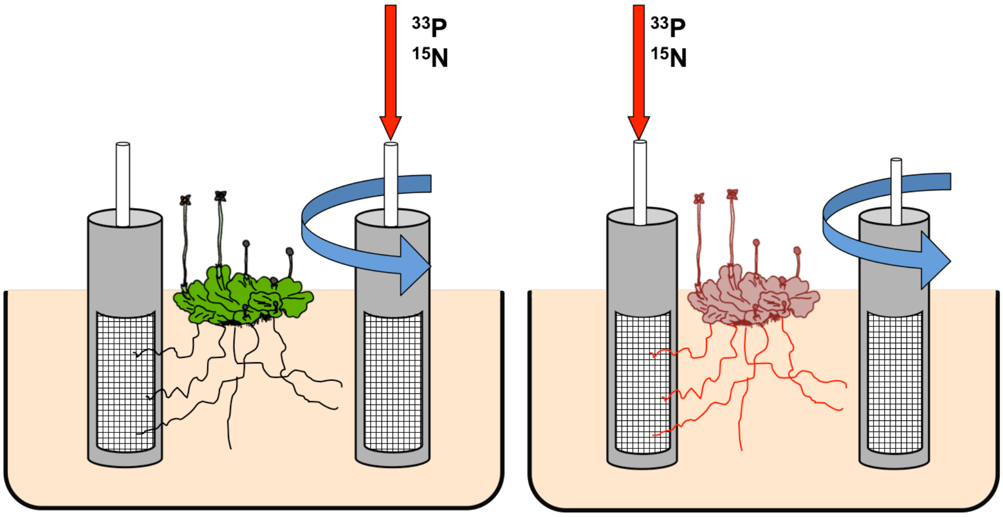

B

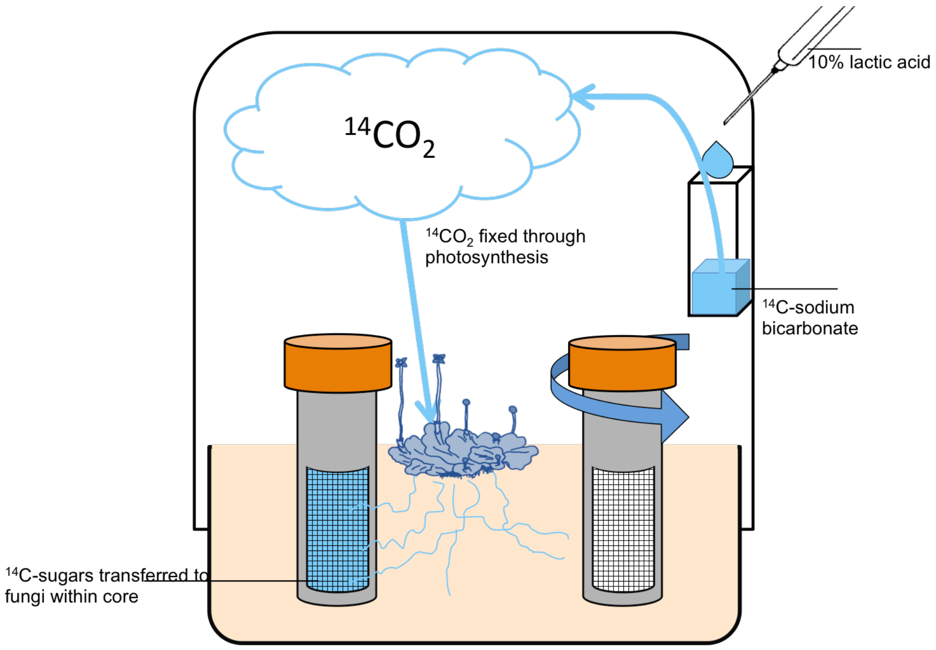

Figure S2. Illustration of experimental procedure for labelling with (a) ^{33}P-orthophosphate and (b) $^{14}CO_2$.

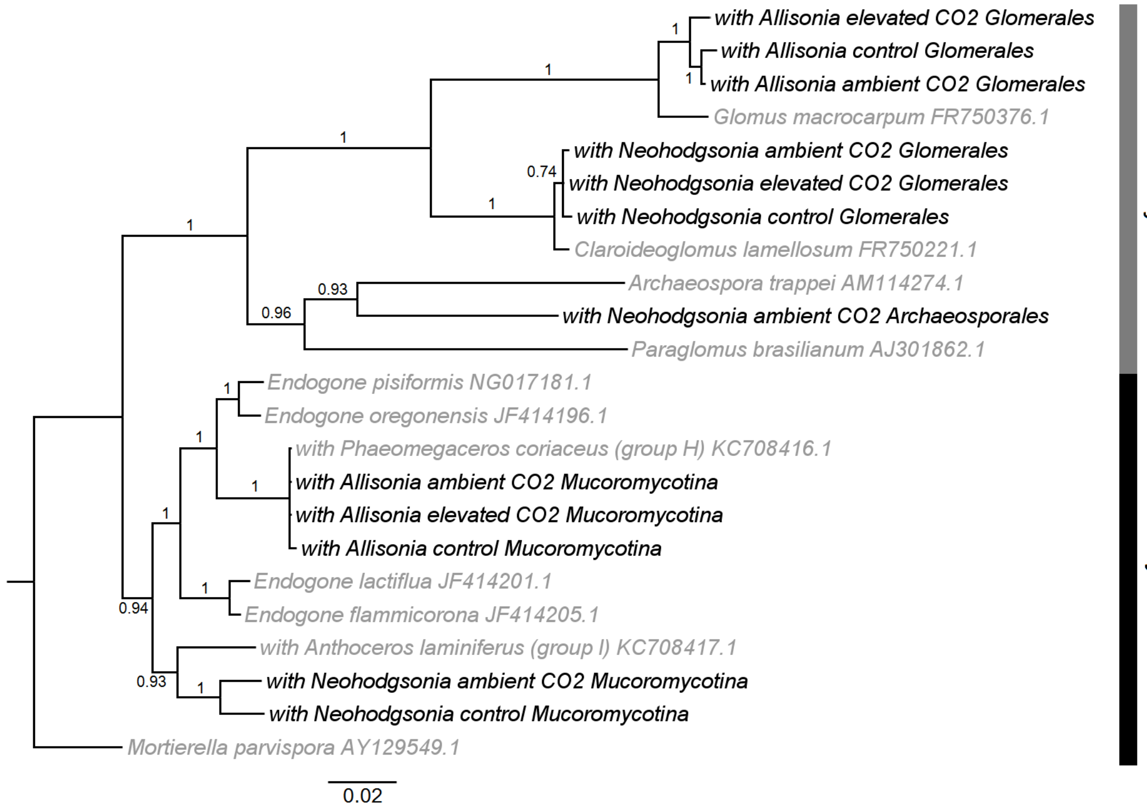

Figure S3. Phylogenetic tree showing the placement of mutualistic fungi colonising *Allisonia cockaynii* and *Neohodgsonia mirabilis*. The 18S ribosomal gene was sequenced using molecular cloning. Control plants were prepared for sequencing within one day of collection. Experimental plants (ambient or elevated CO_2) were prepared for sequencing when plants were harvested at the end of the isotope labelling experiments. New sequences are highlighted in black while reference sequences from GenBank are in grey. Bayesian analysis was performed using an HKY85 model and invgamma rates with four simultaneously run heated chains (chain length: 1.1×10^6). The sequence of Mucoromycotina fungi from *Neohodgsonia* grown under elevated CO_2 is not included in this tree as only a 450bp segment was produced.

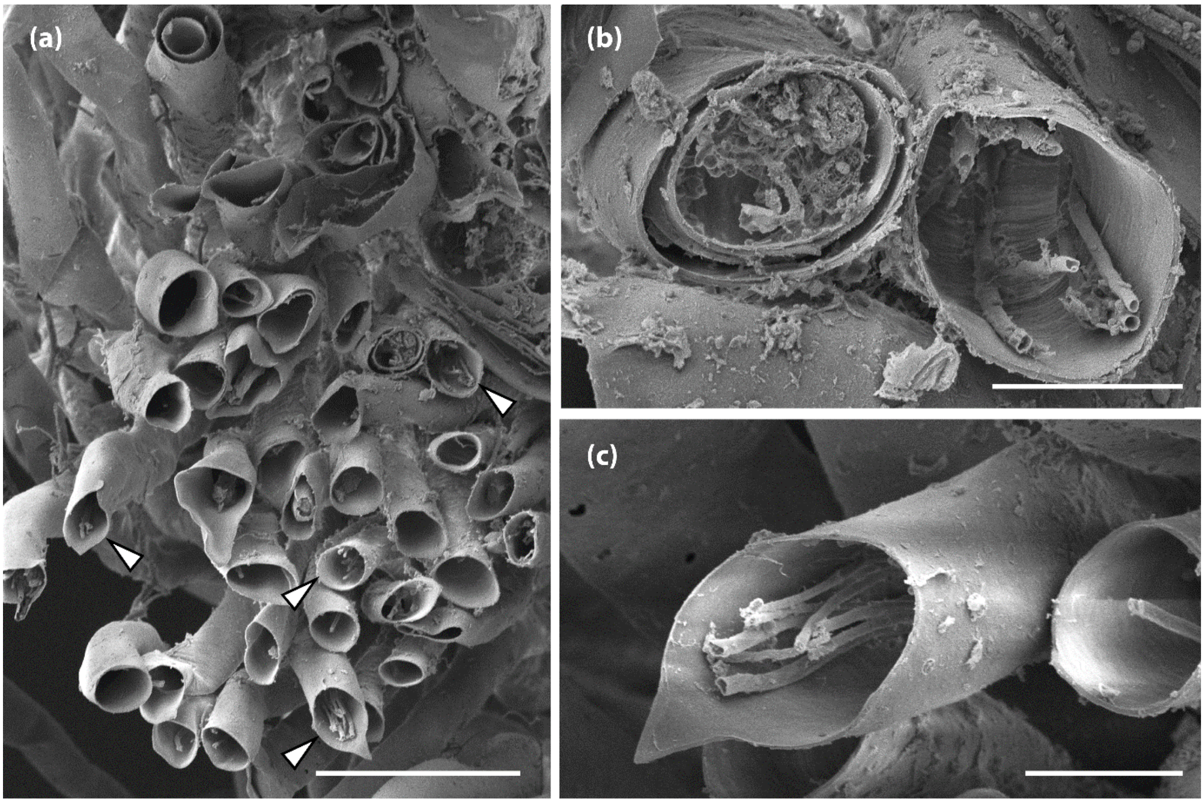

Figure S4. *Neohodgsonia mirabilis*. Scanning electron micrographs. (a) Fungal entry in the basal thalloid liverworts *Neohodgsonia* (complex) and *Allisonia cockaynei* (simple – not shown) is via the rhizoids. Note the numerous fungal hyphae in the rhizoid lumina (arrowed), enlarged in (b) and (c). Scale bars: (a) 100 µ; (b, c) 20 µm.

References

Cameron DD, Johnson I, Leake JR, Read DJ. (2007). Mycorrhizal acquisition of inorganic phosphorus by the green-leaved terrestrial orchid *Goodyera repens*. Ann Bot (London) 99:831-834.

Cameron DD, Leake JR, Read DJ. (2006). Mutualistic mycorrhiza in orchids: evidence from plant-fungus carbon and nitrogen transfers in the green-leaved terrestrial orchid *Goodyera repens*. New Phytol 171:405-416.

Desiró A, Duckett JG, Pressel S, Villarreal JC, Bidartondo MI. (2013). Fungal symbioses in hornworts: a chequered history. Proc Roy Soc B 280:20130207.

Fletcher BJ, Brentnall SJ, Quick WP, Beerling GJ. (2006). BRYOCARB: A process-based model of thallose liverwort isotope fractionation in response to CO_2, O_2, light and temperature. Geochim Cosmochim Ac 70:5676-5691.

Gardes M, Bruns TD. (1993). ITS primers with enhanced specificity for basidiomycetes - application to the identification of mycorrhizae and rusts. Mol Ecol 2:113-118.

Nobel PS. (1999). Physicochemical and Environmental Plant Physiology. 2nd edition. Academic Press.

Smit E, Leeflang P, Glandorf B, van Elsas JD, Wernars K. (1999). Analysis of fungal diversity in the wheat rhizosphere by sequencing of cloned PCR-amplified genes encoding 18S rRNA and temperature gradient gel electrophoresis. Appl Environ Microbiol 65:2614-2621

White TJ, Bruns T, Lee S, Taylor J. (1990). Amplification and direct sequencing of fungal ribosomal RNA genes for phylogenetics. Chapter in: Innis, MA, Gelfand, DH, Sninsky, JJ, White, TJ (eds) PCR Protocols: a Guide to Methods and Applications Academic Press: London, pp 315-322.
